# Abnormalities of Serum Fatty Acids in Children With Henoch–Schönlein Purpura by GC-MS Analysis

**DOI:** 10.3389/fped.2020.560700

**Published:** 2021-01-21

**Authors:** Min Wen, Shipin Feng, Xiqiang Dang, Xuewei Ding, Zhiquan Xu, Xiaoyan Huang, Qiuyu Lin, Wei Xiang, Xiaoyan Li, Xiaojie He

**Affiliations:** ^1^Department of Pediatrics, The Second Xiangya Hospital, Central South University, Changsha, China; ^2^Laboratory of Pediatric Nephrology, Institute of Pediatrics, Central South University, Changsha, China; ^3^Department of Pediatric Nephrology, Chengdu Women's and Children's Central Hospital, School of Medicine, University of Electronic Science and Technology of China, Chengdu, China; ^4^Hainan Maternal and Children's Medical Center, Haikou, China

**Keywords:** Henoch–Schönlein Purpura, fatty acid, medium-and long-chain fatty acid, children, GC-MS

## Abstract

**Purpose:** The objectives of this work were to test the levels of serum medium- and long- chain fatty acids (MLCFAs) in children and to discover their possible relationship with Henoch-Schönlein Purpura (HSP), also known as Immunoglobulin A vasculitis.

**Methods:** A total of 57 children with HSP (HSP group) and 28 healthy children (CON group) were recruited for this study. Serum specimens were collected to detect the compositions and contents of MLCFAs by gas chromatography with mass spectrometry (GC-MS) analysis.

**Results:** The contents of all detected 37 MLCFAs in the HSP group were higher than the healthy group. Thirty-one species of MLCFAs were discovered to have a significant difference (*p* < 0.05) in two groups. Comparing to healthy controls, there were 31, 31, 18 fatty acids showed a statistical difference in the untreated group, regular treated group, and withdrawal group of HSP, respectively. The trend of fatty acids in the three HSP groups was similar to the healthy controls, as well as the untreated group and regular treated group changed more obviously than the withdrawal group. Almitate (C16:0) and 18 carbon atoms (C18) of fatty acids were abundant in all three HSP groups, divided according to the treatment of glucocorticoid. Some fatty acids were found having considerable differences (*p* < 0.05) in three groups. Monounsaturated fatty acids (MUFAs), including elaidate (C18:1T), cis-11,14,17-eicosatrienoic acid ester (C20:1), and cis-15-tetracosenoate (C24:1), were distinctly higher in HSP children with renal damage.

**Conclusion:** Our study revealed that the abnormalities in MLCFA may be associated with the development of HSP. Another interesting finding was that fatty acids contents were changing during the glucocorticoid treatment. Meanwhile, long-chain MUFAs may have an impact on renal damage in HSP patients. Further studies need to be carried out in order to explore the specific mechanism of fatty acids in the course of HSP.

## Introduction

Henoch-Schönlein Purpura (HSP), also known as Immunoglobulin A vasculitis, is a systemic IgA immune complex-mediated vasculitis (1). The primary clinical manifestations of HSP include palpable purpuric rashes in the extremities (especially the lower extremities), arthritis, gastrointestinal symptoms, and renal damage (2, 3). The disease most commonly affects children, especially 2- to 8-year-olds. Although its pathogenesis remains unclear, multiple etiologies, including genetic background and environmental factors, have been suggested to contribute to the onset of HSP (4). The diagnosis is challenging due to relatively variable and heterogeneous symptoms and the lack of specific clinical indicator.

Fatty acids, as main elements of biological membranes, are reported to have effects on inflammation, vascular function, and thrombosis (5) in the progression of many disease, such as systemic lupus erythematosus (6). Medium- and long-chain fatty acids (MLCFAs) include saturated fatty acids (SFAs), monounsaturated fatty acids (MUFAs), and polyunsaturated fatty acids (PUFAs), playing an important role in autoimmune disease as well as nephrotic disorder (7, 8). Such as omega-3 fatty acids, having a variety of anti-inflammatory and immune-modulating effects that may be of relevance to atherosclerosis (9). Hence, this study seeks to obtain data of serum MLCFAs in children with HSP, which will help to address research gaps between HSP and MLCFAs.

## Materials and Methods

### Research Objects and Blood Sample Collection

Fifty-seven patients with HSP in Second Xiangya Hospital of Central South University and 28 healthy children from the same region were recruited for this study. All the HSP patients were younger than 18 years old and met the HSP diagnostic criteria recognized by American Rheumatology Association and EULAR/PreS (10), which is palpable purpuric rashes in the extremities, particularly the lower extremities (necessary) with any of the following: (1) diffuse abdominal pain; (2) biopsy showing significant IgA deposition; (3) acute arthritis or joint pain in any joint; (4) manifestations of kidney damage [hematuria and (or) proteinuria]. The healthy control group (CON group) consisted of healthy children without any disease or drug-using histories in recent 6 months, confirming by screening questionnaires. Ethical approval for this study was obtained from Ethics Committee of the Second Xiangya Hospital of Central South University. Written informed consent was obtained from parents before the study.

We gathered basic information such as age, height, weight, and recent diets of the two groups. Clinical symptoms, laboratory data of blood routine test, renal function, and drug-using history were collected in HSP patients, who were divided into three groups, the untreated group (diagnosed as HSP for the first time and has not been treated with any immunotherapy drugs), the regular treated group (used glucocorticoids with or without immunotherapy drugs of tacrolimus, cyclosporine, and cyclophosphamide), and the withdrawal group (completely discontinued with any drugs for at least 3 months and did not show clinical symptoms). The glucocorticoids treatment was oral prednisone at 0.5–1 mg/kg/d (maximum 60 mg), then gradually reduced the dose after 1 week or intravenous methylprednisolone at 1–1.5 mg/kg/d. At the same time, HSP patients were divided into two groups depending on the renal involvement, including hematuria and (or) proteinuria.

Heparin anticoagulated tubes were used to collect 3 ml whole blood samples from all study objects in the early morning after overnight fasting. Centrifuge at 1,300–2,000 g at 4°C for 10 min and take the upper plasma (not <0.3 ml), which were quickly frozen in liquid nitrogen before storing at −80°C to wait for testing.

### Standard Preparation

Nine mixed standard concentration gradients of 0.5, 5, 10, 25, 50, 100, 250, 500, 1,000, 2,500 mg/L were prepared from 40 kinds of fatty acid methyl ester mixed standard solution, where the concentration was the sum of every component, each made up 2 or 4% of total concentration.

### Metabolite Extraction

The plasma samples were thawed in ice, and 150 μL of them were taken into the centrifuge tube. Adding 6 mL dichloromethane-methanol solution, vortexed for 2 min prior to shaking for 20 min at room temperature. Once the samples were centrifuged at 2,000 rpm for 10 min, transferred the lower chloroform phase to a new glass test tube. Three milliliters of n-hexane and 0.25 μL of internal standard (C19, 10 mg/mL) were added for extraction. Vortex for 2 min, then add 3 mL KOH/methanol (0.4 mol/L). Following standing and layering, took the supernatant to a new glass test tube to blow dry with nitrogen, and added 200 μL n-hexane when vortexed for 2 min. After standing and layering, took the supernatant to a sample bottle.

### GC-MS Analysis

The samples were separated on an Agilent DB-WAX capillary column (30 m × 0.25 mm ID × 0.25 μm) gas chromatography system. The temperature programming was as follows: the initial temperature was 50°C for 3 min, and then increased at 10°C/min up to 220°C and remained there for 20 min. Finally, rising 15°C per minute, it peaked in 250°C for 10 min. The carrier gas was helium, whose velocity was 1.0 mL/min. A QC sample was used for testing and evaluating the stability and repeatability of the system. An Agilent 6890N/5975B gas chromatography-mass spectrometer was used for analysis. The temperatures of the injection port and transmission line were 280 and 250°C, respectively. The electron bombardment ionization (EI) source, SIM scanning mode, and electron energy were 70 eV.

### Statistical Analysis

Using Mass Hunter software, the chromatographic peak area and retention time were extracted to draw a curve and calculate the content of long-chain fatty acids in the sample. Significance levels were set at the 1% level using the student *t*-test to identify differences in metabolite levels.

## Results

### Clinical Characteristics of the Patients

A total of 57 children with HSP (28 males, 29 females) and 28 healthy children (14 males, 14 females) were recruited for this study. The mean age of HSP group was 9.17 ± 3.08 years, and that of the CON group was 10.60 ± 3.66 years (Table 1), showing no significant difference (*p* = 0.061), while body mass index (BMI) of HSP patients were statistically higher (*p* = 0.03). The main clinical symptoms in patients with HSP included palpable rashes in the extremities (48.28%), arthritis (20.70%), gastrointestinal symptoms (20.70%), and renal damage (53.45%). According to drug-using history of glucocorticoids, they were divided into three groups, the untreated group (25.86%), the regular treated group (51.72%), and the withdrawal group (22.41%). Twenty-three HSP patients had proteinuria or (and) hematuria, and 17 did the renal biopsy (29.31%). The results of blood routine and renal function in all patients were collected for correlation analysis (Table 1).

**Table 1:** Clinical characteristics of the patients.

**Characteristic**	**Group**	
	**HSP (*n* = 57)**	**CON (*n* = 28)**
Age (year)	9.17 ± 3.08	10.60 ± 3.66
BMI (kg/m^2^)	16.50 ± 2.85	18.57 ± 4.57
Rashes (*n*)	28 (48.28%)	
Abdominal pain (*n*)	12 (20.70%)	
Arthritis or joint pain (*n*)	12 (20.70%)	
Hematuria (*n*)	20 (34.48%)	
Proteinuria (*n*)	13 (22.41%)	
Kidney biopsy (*n*)	17 (29.31%)	
Untreated group (*n*)	15 (25.86%)	
Regular treated group (*n*)	30 (51.72%)	
Withdrawal group (*n*)	13 (22.41%)	
Blood urea nitrogen (mmol/l)	4.44 ± 1.84	
Serum creatinine (μmol/l)	40.78 ± 18.87	
Uric acid (μmol/l)	268.95 ± 67.81	
White blood cell (10^9^/l)	8.88 ± 2.69	
Leukocyte (%)	60.3 ± 13.2	
Lymphocyte (%)	32.1 ± 12.5	
Red blood cell (10^12^/l)	4.70 ± 0.47	
Hemoglobin (g/l)	129 ± 12.3	
Platelet (10^9^/l)	310 ± 86.9	

*All values presented as number (%) or mean ± standard deviation. p-value of comparison for age between HSP and CON group was 0.061; p-value of comparison for BMI between HSP and CON group was 0.03*.

### Identification of MLCFAs

**Figure 1:**
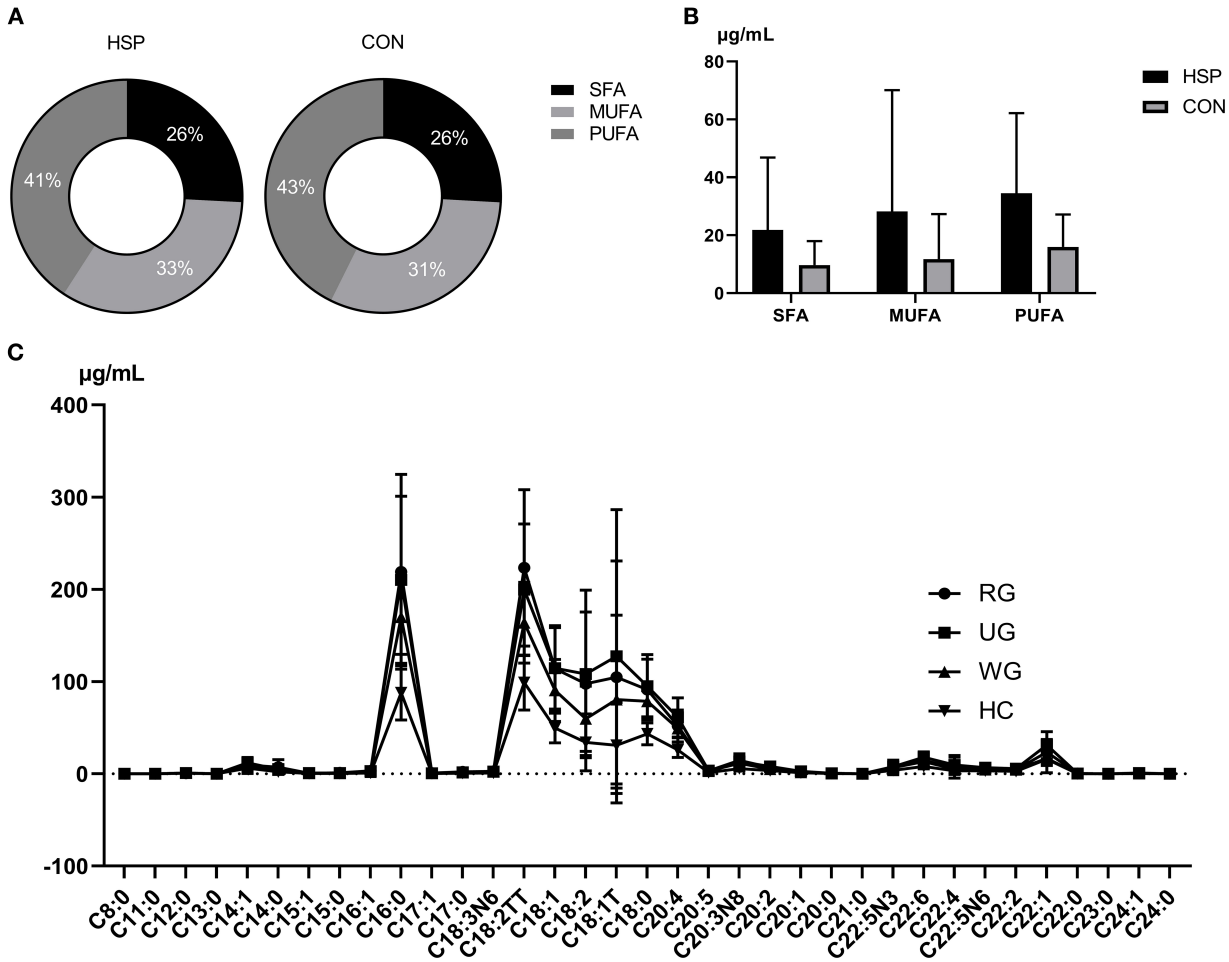
Percent **(A)** and content **(B)** comparison of SFAs, MUFAs, and PUFAs between HSP group and CON group. **(C)** Distribution and correlations of 35 fatty acids among three groups of HSP and CON group. RG means regular treated group, UG means the untreated group, WG means the withdrawal group. Data are means ± SD.

Forty fatty-acid methyl ester standards were analyzed using the established fatty acid analysis method, and a total of 35 fatty acids were detected and quantified in this experiment (Table 2, Supplementary Table 1), of which 1 fatty acid was not separated due to its isomers, and 4 were not detected in all samples. The internal standard was separated from each standard, and the chromatographic separation of each metabolite is ideal with sharp and symmetrical peak shape, which make it possible to quantify each metabolite by mass spectrometry. The linearity and correlation coefficient for each component, representing the proportion of each component in the total concentration, is >0.99 and showed that the linearity of each analyte is good. Comparing with healthy children, all detected fatty acids were upregulated in HSP group, and 31 of them obviously increased (*p* < 0.05), which includes 11 kinds of SFAs, 8 kinds of MUFAs and 12 kinds of PUFAs. No obvious differences were found between the percent of SFA, MUFA, PUFA in the two groups (Figure 1A), but the contents in HSP patients were all higher than CON group (Figure 1B). Among them, the enhance of decanoate (C10:0), behenate (C22:0), and etracosanoate (C24:0) were relatively more conspicuous (fold change > 4).

**Table 2:** The percentage composition of 35 free fatty acids found in plasma from patients with HSP group and CON group.

**Component name**	**MLCFAs**	**Mean ± SD**		**Fold change**	***p*-value**
		**HSP (*n* = 57)**	**CON (*n* = 28)**		
***SFA***					
C8:0	octanoate	0.034 ± 0.002	0.017 ± 0.001	1.958	<0.001
C11:0	undecanoate	0.013 ± 0.007	0.007 ± 0.003	1.773	<0.001
C12:0	dodecanoate	0.737 ± 0.713	0.648 ± 1.212	1.137	0.672
C13:0	tridecanoate	0.006 ± 0.011	0.003 ± 0.006	2.111	0.146
C14:0	myristate	6.026 ± 6.669	2.085 ± 1.947	2.89	0.003
C15:0	pentadecanoate	0.922 ± 0.897	0.283 ± 0.206	3.254	<0.001
C16:0	palmitate	205.706 ± 91.747	87.878 ± 29.930	2.341	<0.001
C17:0	thyl heptadecanoate	1.958 ± 1.535	0.701 ± 0.316	2.795	<0.001
C18:0	stearate	89.292 ± 30.546	43.354 ± 12.192	2.06	<0.001
C20:0	arachidate	0.516 ± 0.392	0.239 ± 0.192	2.162	0.001
C21:0	heneicosanoate	0.016 ± 0.018	0.006 ± 0.007	2.573	0.009
C22:0	behenate	0.399 ± 0.743	0.080 ± 0.100	4.998	0.025
C23:0	tricosanoate	0.022 ± 0.020	0.009 ± 0.011	2.371	0.002
C24:0	tetracosanoate	0.172 ± 0.415	0.037 ± 0.048	4.698	0.09
***PUFA***					
C18:3N6	γ-linolenate	2.561 ± 1.802	0.865 ± 0.344	2.961	<0.001
C18:2TT	linolelaidate	203.965 ± 76.713	98.960 ± 30.316	2.061	<0.001
C18:2	linoleate	91.650 ± 75.122	34.114 ± 31.357	2.687	<0.001
C20:4	arachidonate	53.559 ± 17.360	25.960 ± 8.396	2.063	<0.001
C20:5	cis-5,8,11,14,17-Eicosapentaenoic acid ester	3.318 ± 1.895	1.344 ± 0.677	2.469	<0.001
C20:3N8	cis-8,11,14-Eicosatrienoic acid ester	13.638 ± 6.174	5.524 ± 2.525	2.469	<0.001
C20:2	cis-11,14-Eicosadienoic acid ester	6.748 ± 3.213	3.571 ± 1.182	1.89	<0.001
C22:5N3	docosapentaenoate	6.965 ± 6.739	3.845 ± 3.243	1.812	0.023
C22:6	cis-4,7,10,13,16,19-Docosahexaenoic acid ester	14.752 ± 5.598	7.966 ± 2.668	1.852	<0.001
C22:4	docosatetraenoate	6.538 ± 8.192	3.158 ± 2.456	2.07	0.036
C22:5N6	docosapentaenoate	5.909 ± 3.203	3.266 ± 2.443	1.809	<0.001
C22:2	cis-13,16-Docosadienoic acid ester	4.709 ± 3.215	2.777 ± 1.221	1.696	0.003
***MUFA***					
C14:1	myristoleate	9.450 ± 5.816	6.053 ± 2.747	1.561	0.004
C15:1	cis-10-pentadecenoate	0.715 ± 0.439	0.445 ± 0.262	1.606	0.004
C16:1	palmitoleate	2.835 ± 1.800	0.974 ± 0.425	2.911	<0.001
C17:1	cis-10-heptadecenoate	0.825 ± 0.815	0.222 ± 0.138	3.718	<0.001
C18:1	oleate	108.742 ± 43.763	49.710 ± 16.429	2.188	<0.001
C18:1T	elaidate	104.847 ± 127.147	30.928 ± 47.252	3.39	0.004
C20:1	cis-11,14,17-Eicosatrienoic acid ester	2.183 ± 1.303	1.074 ± 0.414	2.032	<0.001
C22:1	erucate	23.939 ± 14.932	15.888 ± 6.963	1.507	0.008
C24:1	cis-15-tetracosenoate	0.614 ± 1.255	0.335 ± 0.421	1.832	0.257

*Fold Change: multiples of change of metabolites in the comparison group, >1 indicates up-regulation, and <1 indicates down-regulation; p-value: statistical analysis of metabolites in the comparison group, p < 0.05 is a significant difference in metabolites*.

### Compositions and Correlations of Fatty Acids in Different Groups of HSP and CON Group

The distribution of 35 serum fatty acids in three groups of HSP was presented in Figure 1C. Comparing to healthy controls, there were 31, 31, and 18 fatty acids showed a statistical difference in the untreated group, regular treated group, and withdrawal group of HSP, respectively (Supplementary Table 2). The trend of fatty acids in the three HSP groups was similar to the healthy controls, as well as the untreated group and regular treated group changed more obviously than the withdrawal group. As the figure shows, almitate (C16:0) and C18 were abundant in all three HSP groups. The difference between three groups is analyzed using analysis theory of mathematical statistics, and statistical significance can be seen in erucate (C22:1) and docosahexaenoic acid (DHA, C22:6). A series of fatty acids were distinctly downregulated in the withdraw group in contrast to the untreated group, including undecanoate (C11:0), myristoleate (C14:1), cis-10-pentadecenoate (C15:1), cis-11,14-eicosadienoic acid ester (C20:2), eicosatrienoic acid ester (C20:1), docosapentaenoate (C22:5N3), and erucate (C22:1). DHA (C22:6) was significantly downregulated in the regular treated group compared with the untreated group. Failed to pass homogeneity test of variance, the compare between the regular treated group and the withdrawal group is carried out by means of Wilcoxon rank sum test, exerting that palmitoleate (C16:1), linolelaidate (C18:2TT), and tetracosanoate (C24:0) were statistically higher in the former group.

### Correlation Analysis With Laboratory Test Results of HSP

**Figure 2:**
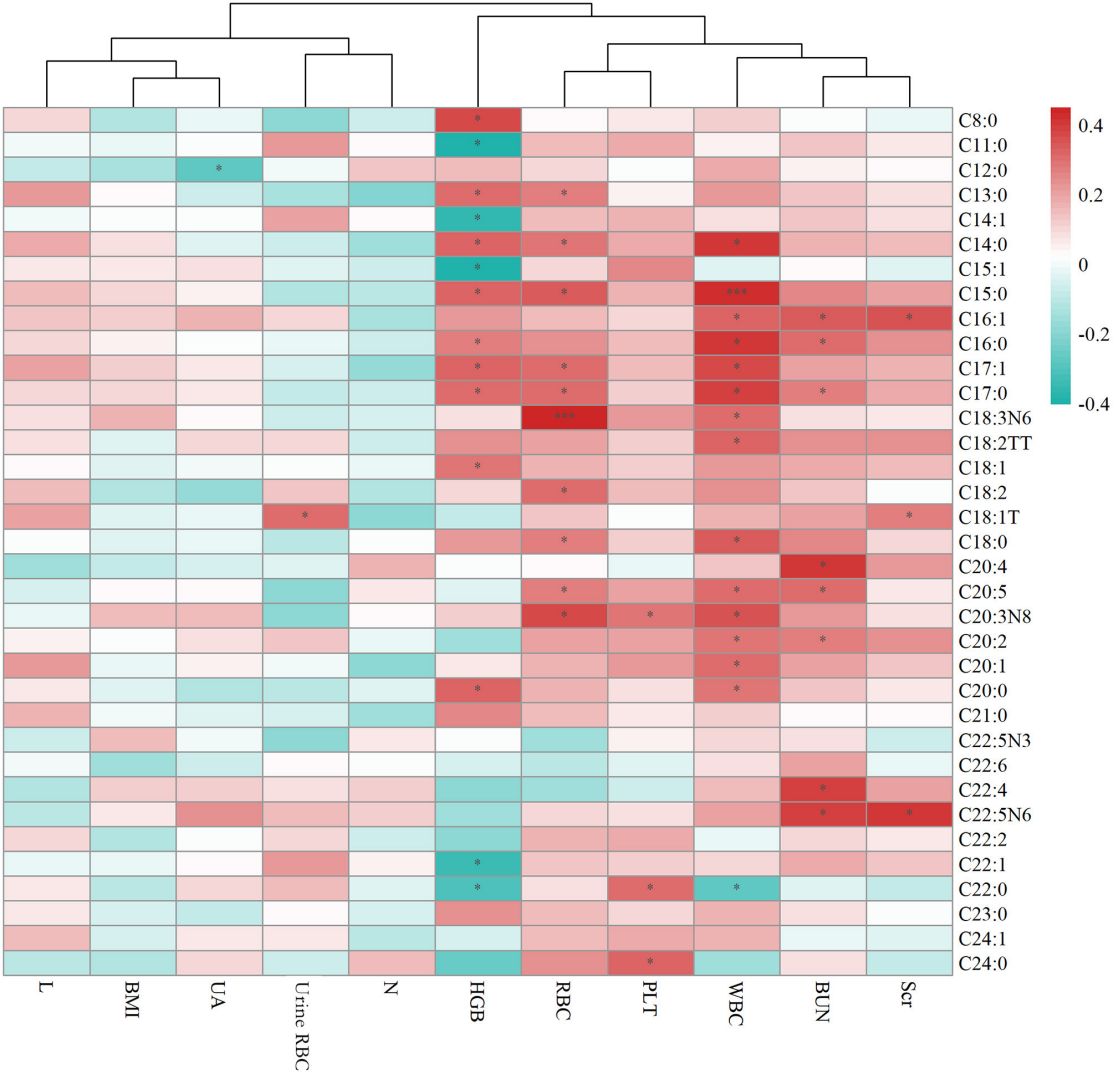
Heat map summarizing the correlation between serum fatty acids and laboratory test results of HSP. Scr means Serum creatinine (umol/l), BUN means Blood urea nitrogen (mmol/l), UA means Uric acid (umol/l), WBC means White blood cell (10^9^/l), RBC means Red blood cell (10^12^/l), PLT means Platelet (10^9^/l), HGB means Hemoglobin (g/l), N means the percent of Leukocyte, L means the percent of Lymphocyte, BMI shows Body Mass Index. * means *p* < 0.05, *** means *p* < 0.001.

Figure 2 displays the correlation between plasma metabolites and test results of HSP. It is apparent from this graph that there were positive correlations between free fatty acids and clinical indicators, such as WBC and C15:0 (*p* < 0.001) as well as RBC and γ-linolenate (*p* < 0.001).

### Relationship Between Serum Fatty Acids and Renal Damage of HSP

**Figure 3:**
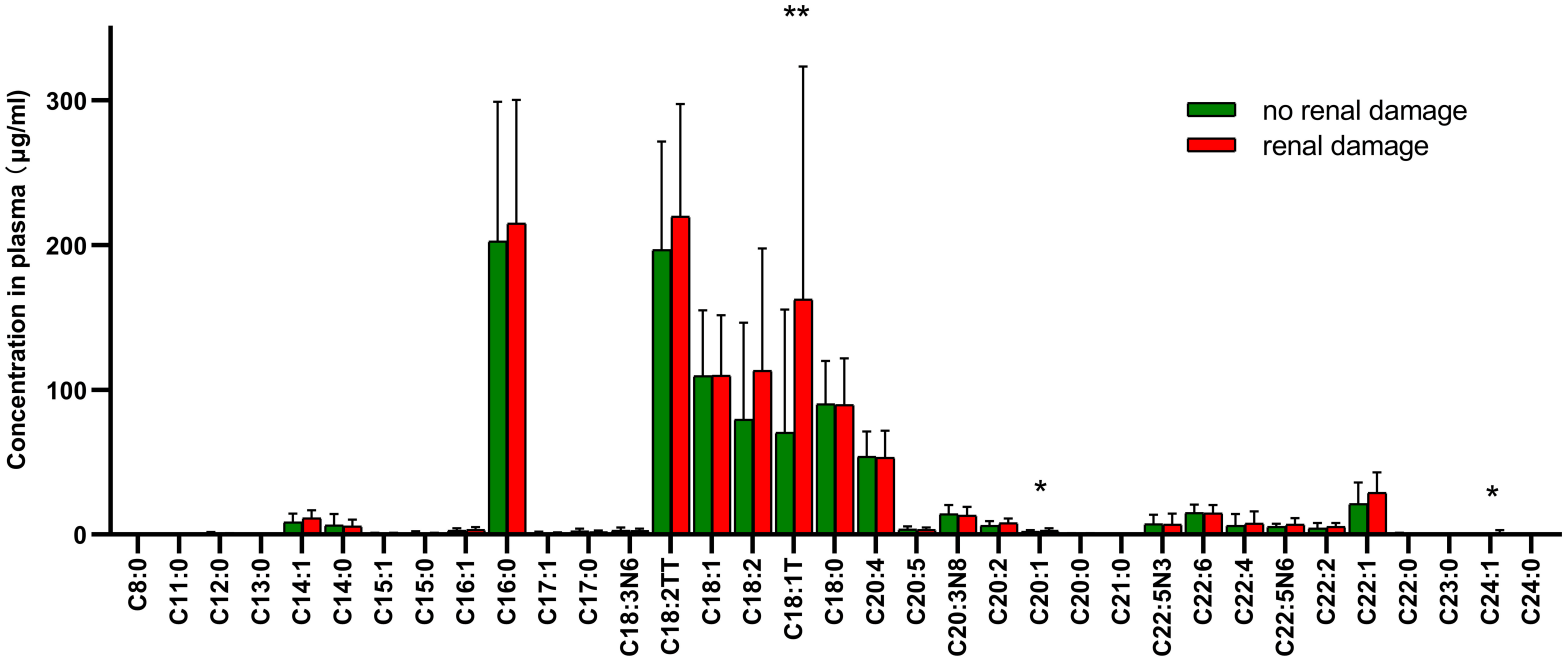
Distributions and correlation in the compositions of fatty acids in two groups with or without renal damage. * means *p* < 0.05, ** means *p* < 0.01.

The patients of HSP were divided into two groups according to whether they had proteinuria or (and) hematuria. Figure 3 showed the compositions of fatty acids in two groups with significant difference in C18:1T, C20:1, and C24:1, which are all MUFAs and were more abundant in the group with renal impairment than that in the group without renal impairment.

## Discussion

In our study, the different kinds of free fatty acids were isolated and analyzed by GC-MS (11), which is a method that combines the characteristics of gas chromatography and mass spectrometry to identify different substances in a blood or urine sample when used in medical research (12, 13). Diseases such as systemic lupus erythematosus (6), search for metabolic markers in this way. Thirty-one fatty acids in our HSP group were detected to be differentially upregulated in the plasma compared to the control group, containing both saturated fatty acids and unsaturated fatty acids, suggesting upregulated fatty acid catabolism. The changes of MLCFAs in untreated group and regular treated group are more obvious than withdrawal group, suggesting that it is the disease itself rather than immunomodulatory treatment caused the differences observed between HSP and CON groups. This observation may support the possible role of MLCFAs we have detected in the pathogenesis of HSP through the well-known fatty acid metabolism. A note of caution is due here since there are limited studies having investigated the relationship between fatty acids and the development of HSP.

Generally, the common SFAs (14) include caprylic acid, lauric acid, myristic acid, palmitic acid, stearic acid, arachidic acid, etc. Palmitic acid was found to be able to stimulate the release of extracellular vesicles from proximal tubular epithelial cells, resulting in kidney dysfunction in the context of metabolic disease (15). MUFAs like oleic acid have the ability to regulate cholesterol metabolism. Long-chain MUFAs have been shown to attenuate atherosclerosis development in mouse models. Yang et al. (16) found C20:1 or erucate (C22:1) was equally effective in reducing atherosclerosis in LDLr mice through activation of the Ppar signaling pathways and favorable alterations in the proteome of lipoproteins. In the current study, we found C18:1T, C20:1, and C24:1 were significantly higher in the group with renal damage than that in the group without renal impairment, indicating long-chain MUFAs may have an interaction with renal function. C18:1T also had a correlation with urine red blood as Figure 2 shows. These results corroborate the findings a study of fatty acid in chronic kidney disease, which discovered increased concentrations of MUFAs by activating of the palmitic and oleic acid pathway and defined the index C18:3n6/C22:4n6 as a new marker of chronic kidney disease (17). PUFAs can be classified into omega-3 and omega-6 polyunsaturated fatty acids depending on the position of the first double bond from the methyl end. DHA and Eicosapentaenoic acid (EPA) are two most important omega-3 PUFAs for the human body, and are both able to ameliorate renal disease (18). In addition, they have effects of anti-atherosclerotic, reducing blood lipids, and inhibiting platelet aggregation. DHA can also effectively reduce the activity of inflammatory factors mainly by inhibiting the 5-lipoxygenase metabolism pathway of neutrophils and monocytes and increase the synthesis of leukotriene B5. Arachidonic acid (AA), a kind of omega-6 PUFA, mainly manifests as the form of phospholipids in the cell membrane, and is released from the phospholipids under stress by phospholipase A2 and phospholipase C (7). AA is of great importance in a series of physiological activities such as esterifying cholesterol, increasing vascular elasticity, reducing blood viscosity, and regulating blood cell function. It also synthesizes bioactive substances of prostaglandins, thromboxanes and leukotrienes, contributing to the immune system (19). All the fatty acids mentioned above were up-regulated in HSP group, indicating that fatty acid metabolism may play an important role in the course of HSP, but the specific mechanism remains unknown, requiring further research. To develop a full picture, our research group will continue studying the role of different types of fatty acids in the pathogenesis of HSP by *in vitro* and animal experiments.

So far, there are no specific laboratory indicators detected in the disease of HSP. There are some significant correlations between free fatty acids and clinical indicators, which are considered to be related to the biofilm membrane lipid composition. Some kinds of fatty acids found in our study having changes according to the treatment, which may suggest that fatty acid metabolism played a significant role in the improvement of HSP. All of the patients in the regular treated group used glucocorticoids (methylprednisolone or prednisone), while parts of the patients used both glucocorticoids and other immune-suppressors such as tacrolimus, cyclophosphamide, and Mycophenolate mofetil. Previous reviews have shown glucocorticoids are important determinants of fatty acid metabolism in both animals and humans (20). A finding of Stephanie Tung et al. (21) showed fatty acid oxidation may take part in the mechanism of resistance to glucocorticoid-mediated cytotoxicity, and PPARα inhibition may improve the therapeutic efficacy of glucocorticoids. There is abundant room for further progress in dissecting the integrated effects on fatty acid and glucocorticoid metabolism.

Last, it is interesting to note that there was a significant difference in BMI between HSP group and healthy controls of the same age (*p* = 0.03). HSP patients tend to have a lower BMI despite normal diets. BMI is an indicator closely related to fatty acid metabolism (22). In previous studies of many diseases, such as diabetes, the important role of BMI in fat metabolism has been observed (23). This outcome is contrary to some studies about HSP that did not show the difference between HSP group and healthy patients in BMI, or length as well as weight (24). Although HSP is currently considered to be an acute attack disease, its pathogenesis may be related to disorders of fatty acid metabolism and weight loss. Yet, whether and how HSP is related to BMI remain to be an important issue for future research.

## Conclusions

Our study provides clinical evidence to support that MLCFA metabolism is associated with HSP by GC-MS method. Glucocorticoid therapy may have a close relationship with fatty acid metabolism during HSP treatment. Meanwhile, long-chain MUFAs may have an impact on renal damage of HSP. Further studies are needed to explore the specific mechanism of fatty acids and HSP.

## Data Availability Statement

The raw data supporting the conclusions of this article will be made available by the authors, without undue reservation.

## Ethics Statement

The studies involving human participants were reviewed and approved by Ethics Committee of the Second Xiangya Hospital of Central South University. Written informed consent to participate in this study was provided by the participants' legal guardian/next of kin.

## Author Contributions

All authors listed have made a substantial, direct and intellectual contribution to the work, and approved it for publication.

## Conflict of Interest

The authors declare that the research was conducted in the absence of any commercial or financial relationships that could be construed as a potential conflict of interest.
