# Supplementary material for: Abnormalities of Serum Fatty Acids in Children With Henoch–Schönlein Purpura by GC-MS Analysis
Source: Front Pediatr. 2021 Jan 21;8:560700. doi: 10.3389/fped.2020.560700 (PMC7860144; doi:10.3389/fped.2020.560700)
Supplement: Supplementary file 2 [file Table_2.docx]

**Supplementary Table 2:Different free fatty acids among three groups respectively with CON group**

**(1) Different free fatty acids found in plasma from patients with RG and CON group**

| Component name | MLCFAs | Mean ± SD | | Fold Change | p value |
| --- | --- | --- | --- | --- | --- |
|  |  | RG(n=30) | CON(n=28) |  |  |
| C8:0 | octanoate | 0.035±0.003 | 0.017±0.001 | 1.980936 | <0.001 |
| C11:0 | undecanoate | 0.013±0.006 | 0.007±0.003 | 1.765187 | <0.001 |
| C14:1 | myristoleate | 9.505±5.175 | 2.085±1.947 | 1.570116 | <0.001 |
| C14:0 | myristate | 7.290±7.962 | 0.283±0.206 | 3.496211 | 0.043 |
| C15:1 | cis-10-pentadecenoate | 0.699±0.425 | 87.878±29.930 | 1.570525 | <0.001 |
| C15:0 | pentadecanoate | 1.096±1.100 | 0.701±0.316 | 3.86893 | <0.001 |
| C16:1 | palmitoleate | 3.212±2.019 | 43.354±12.192 | 3.297374 | <0.001 |
| C16:0 | palmitate | 219.071±103.747 | 0.239±0.192 | 2.492915 | <0.001 |
| C17:1 | cis-10-heptadecenoate | 0.999±0.991 | 0.006±0.007 | 4.500799 | <0.001 |
| C17:0 | thyl heptadecanoate | 2.295±1.893 | 0.399±0.743 | 3.260767 | <0.001 |
| C18:3N6 | γ-linolenate | 2.784±1.896 | 0.172±0.415 | 3.218186 | <0.001 |
| C18:2TT | linolelaidate | 223.325±83.264 | 0.022±0.020 | 2.256725 | <0.001 |
| C18:1 | oleate | 114.041±45.733 | 0.865±0.344 | 2.294122 | <0.001 |
| C18:2 | linoleate | 97.721±76.450 | 98.960±30.316 | 2.864513 | <0.001 |
| C18:1T | elaidate | 104.874±123.929 | 34.114±31.357 | 3.390865 | <0.001 |
| C18:0 | stearate | 91.301±32.401 | 25.960±8.396 | 2.105923 | <0.001 |
| C20:4 | arachidonate | 51.952±17.022 | 1.344±0.677 | 2.001283 | <0.001 |
| C20:5 | cis-5,8,11,14,17-Eicosapentaenoic acid ester | 3.366±1.792 | 5.524±2.525 | 2.504883 | <0.001 |
| C20:3N8 | cis-8,11,14-Eicosatrienoic acid ester | 14.696±6.980 | 3.571±1.182 | 2.660448 | <0.001 |
| C20:2 | cis-11,14-Eicosadienoic acid ester | 6.989±3.080 | 3.845±3.243 | 1.957183 | <0.001 |
| C20:1 | cis-11,14,17-Eicosatrienoic acid ester | 2.197±1.271 | 7.966±2.668 | 2.045936 | <0.001 |
| C20:0 | arachidate | 0.547±0.359 | 6.538±8.192 | 2.291521 | 0.03 |
| C21:0 | heneicosanoate | 0.020±0.023 | 5.909±3.203 | 3.18416 | 0.002 |
| C22:6 | cis-4,7,10,13,16,19-Docosahexaenoic acid ester | 13.275±5.226 | 6.053±2.747 | 1.666343 | <0.001 |
| C22:4 | DOCOSATETRAENOATE | 5.052±4.630 | 0.445±0.262 | 1.599711 | 0.004 |
| C22:5N6 | DOCOSAPENTAENOATE | 6.159±3.599 | 0.974±0.425 | 1.885752 | <0.001 |
| C22:2 | cis-13,16-Docosadienoic acid ester | 4.309±2.390 | 0.222±0.138 | 1.55175 | <0.001 |
| C22:1 | erucate | 23.673±13.329 | 49.710±16.429 | 1.490048 | <0.001 |
| C22:0 | behenate | 0.445±0.831 | 30.928±47.252 | 5.568703 | 0.02 |
| C23:0 | tricosanoate | 0.025±0.023 | 1.074±0.414 | 2.692716 | 0.004 |
| C24:0 | tetracosanoate | 0.210±0.521 | 0.614±1.255 | 5.736136 | 0.004 |

**(2) Different free fatty acids found in plasma from patients with UG and CON group**

| Component name | | MLCFAs | Mean ± SD | | Fold Change | p value |
| --- | --- | --- | --- | --- | --- | --- |
|  |  |  | UG(n=14) | CON(n=28) |  |  |
| C8:0 | octanoate | | 0.034±0.002 | 0.017±0.001 | 1.950041 | <0.001 |
| C11:0 | undecanoate | | 0.016±0.006 | 0.007±0.003 | 2.187917 | <0.001 |
| C14:1 | myristoleate | | 11.951±5.615 | 2.085±1.947 | 1.974255 | <0.001 |
| C14:0 | myristate | | 4.875±5.131 | 0.283±0.206 | 2.337961 | 0.043 |
| C15:1 | cis-10-pentadecenoate | | 0.903±0.360 | 87.878±29.930 | 2.029161 | 0.001 |
| C15:0 | pentadecanoate | | 0.781±0.622 | 0.701±0.316 | 2.754406 | <0.001 |
| C16:1 | palmitoleate | | 2.911±1.603 | 43.354±12.192 | 2.988587 | <0.001 |
| C16:0 | palmitate | | 87.220±210.388 | 0.239±0.192 | 2.3941 | <0.001 |
| C17:1 | cis-10-heptadecenoate | | 0.697±0.545 | 0.006±0.007 | 3.14207 | <0.001 |
| C17:0 | thyl heptadecanoate | | 1.770±1.017 | 0.399±0.743 | 2.525953 | <0.001 |
| C18:3N6 | γ-linolenate | | 2.465±1.520 | 0.172±0.415 | 2.849453 | <0.001 |
| C18:2TT | linolelaidate | | 199.650±68.764 | 0.022±0.020 | 2.017488 | <0.001 |
| C18:1 | oleate | | 114.403±42.543 | 0.865±0.344 | 2.301395 | <0.001 |
| C18:2 | linoleate | | 108.306±87.620 | 98.960±30.316 | 3.174783 | <0.001 |
| C18:1T | elaidate | | 127.439±153.329 | 34.114±31.357 | 4.120462 | <0.001 |
| C18:0 | stearate | | 94.929±33.041 | 25.960±8.396 | 2.189611 | <0.001 |
| C20:4 | arachidonate | | 61.142±20.461 | 1.344±0.677 | 2.355272 | <0.001 |
| C20:5 | cis-5,8,11,14,17-Eicosapentaenoic acid ester | | 3.516±1.741 | 5.524±2.525 | 2.61644 | <0.001 |
| C20:3N8 | cis-8,11,14-Eicosatrienoic acid ester | | 13,324±5.461 | 3.571±1.182 | 2.412077 | <0.001 |
| C20:2 | cis-11,14-Eicosadienoic acid ester | | 7.882±3.126 | 3.845±3.243 | 2.207255 | <0.001 |
| C20:1 | cis-11,14,17-Eicosatrienoic acid ester | | 2.707±1.554 | 7.966±2.668 | 2.520963 | <0.001 |
| C20:0 | arachidate | | 0.563±0.481 | 6.538±8.192 | 2.357292 | 0.03 |
| C21:0 | heneicosanoate | | 0.015±0.008 | 5.909±3.203 | 2.364446 | 0.001 |
| C22:6 | cis-4,7,10,13,16,19-Docosahexaenoic acid ester | | 18.046±5.663 | 6.053±2.747 | 2.265357 | <0.001 |
| C22:4 | DOCOSATETRAENOATE | | 9.592±9.950 | 0.445±0.262 | 3.037419 | 0.004 |
| C22:5N6 | DOCOSAPENTAENOATE | | 6.731±2.980 | 0.974±0.425 | 2.060928 | <0.001 |
| C22:2 | cis-13,16-Docosadienoic acid ester | | 5.700±2.470 | 0.222±0.138 | 2.053158 | <0.001 |
| C22:1 | erucate | | 31.145±14.139 | 49.710±16.429 | 1.960323 | <0.001 |
| C22:0 | behenate | | 0.459±0.648 | 30.928±47.252 | 5.741356 | 0.02 |
| C23:0 | tricosanoate | | 0.238±0.168 | 1.074±0.414 | 2.528452 | 0.004 |
| C24:0 | tetracosanoate | | 0.197±0.294 | 0.614±1.255 | 5.381981 | 0.004 |

**(3) Different free fatty acids found in plasma from patients with WG and CON group**

| Component name | MLCFAs | Mean ± SD | | Fold Change | p value |
| --- | --- | --- | --- | --- | --- |
|  |  | UG(n=14) | CON(n=28) |  |  |
| C8:0 | octanoate | 0.034±0.001 | 0.017±0.001 | 1.913 | <0.001 |
| C14:0 | myristate | 4.350±3.007 | 2.085±1.912 | 2.086 | 0.029 |
| C15:0 | pentadecanoate | 0.673±0.329 | 0.283±0.202 | 2.374 | <0.001 |
| C16:1 | palmitoleate | 1.884±0.741 | 0.974±0.418 | 1.934 | <0.001 |
| C16:0 | palmitate | 169.823±38.800 | 87.878±29.391 | 1.932 | <0.001 |
| C17:1 | cis-10-heptadecenoate | 0.562±0.341 | 0.222±0.135 | 2.53 | <0.001 |
| C17:0 | thyl heptadecanoate | 1.408±0.429 | 0.701±0.310 | 2.01 | <0.001 |
| C18:3N6 | γ-linolenate | 2.152±1.709 | 0.865±0.338 | 2.487 | 0.002 |
| C18:2TT | linolelaidate | 163.934±42.089 | 98.960±29.769 | 1.657 | <0.001 |
| C18:1 | oleate | 90.417±32.237 | 49.710±16.133 | 1.819 | <0.001 |
| C18:2 | linoleate | 59.703±33.906 | 34.114±30.792 | 1.75 | 0.025 |
| C18:1T | elaidate | 80.453±87.946 | 30.928±46.401 | 2.601 | 0.001 |
| C18:0 | stearate | 78.585±16.208 | 43.354±11.972 | 1.813 | <0.001 |
| C20:4 | arachidonate | 49.098±9.539 | 25.960±8.245 | 1.891 | <0.001 |
| C20:5 | cis-5,8,11,14,17-Eicosapentaenoic acid ester | 2.994±2.157 | 1.344±0.665 | 2.228 | <0.001 |
| C20:3N8 | cis-8,11,14-Eicosatrienoic acid ester | 11.534±3.543 | 5.524±2.480 | 2.088 | <0.001 |
| C20:1 | cis-11,14,17-Eicosatrienoic acid ester | 1.584±0.577 | 1.074±0.406 | 1.475 | 0.003 |
| C22:6 | cis-4,7,10,13,16,19-Docosahexaenoic acid ester | 14.614±4.547 | 7.966±2.620 | 1.834 | <0.001 |
